# Virtual Screening of *C. Sativa* Constituents for the Identification of Selective Ligands for Cannabinoid Receptor 2

**DOI:** 10.3390/ijms21155308

**Published:** 2020-07-26

**Authors:** Mikołaj Mizera, Dorota Latek, Judyta Cielecka-Piontek

**Affiliations:** 1 Department of Pharmacognosy, Poznan University of Medical Sciences, 60-781 Poznań, Poland; mikolajmizera@gmail.com; 2 Faculty of Chemistry, University of Warsaw, 02-093 Warsaw, Poland; dlatek@chem.uw.edu.pl

**Keywords:** QSAR, endocannabinoid system, *Cannabis Sativa*

## Abstract

The selective targeting of the cannabinoid receptor 2 (CB2) is crucial for the development of peripheral system-acting cannabinoid analgesics. This work aimed at computer-assisted identification of prospective CB2-selective compounds among the constituents of *Cannabis Sativa*. The molecular structures and corresponding binding affinities to CB1 and CB2 receptors were collected from ChEMBL. The molecular structures of *Cannabis Sativa* constituents were collected from a phytochemical database. The collected records were curated and applied for the development of quantitative structure-activity relationship (QSAR) models with a machine learning approach. The validated models predicted the affinities of *Cannabis Sativa* constituents. Four structures of CB2 were acquired from the Protein Data Bank (PDB) and the discriminatory ability of CB2-selective ligands and two sets of decoys were tested. We succeeded in developing the QSAR model by achieving Q^2^ 5-CV > 0.62. The QSAR models helped to identify three prospective CB2-selective molecules that are dissimilar to already tested compounds. In a complementary structure-based virtual screening study that used available PDB structures of CB2, the agonist-bound, Cryogenic Electron Microscopy structure of CB2 showed the best statistical performance in discriminating between CB2-active and non-active ligands. The same structure also performed best in discriminating between CB2-selective ligands from non-selective ligands.

## 1. Introduction

The medical effect of *Cannabis* sp. bioactive ingredients is the subject of extensive research [1,2,3]. It has been discovered that *Cannabis* sp. contains over 500 various compounds, with the cannabinoid group itself having about 110 molecules [4]. Phytocannabinoids present in *Cannabis* sp. have slight differences in their chemical structures (types: cannabidiol, cannabichromene, cannabitriol, cannabicycol, cannabinodiol, cannabinol, cannabielsoin, cannabigerol, Δ^9^-tetrahydrocannabinol, Δ^8^-tetrahydrocannabinate) and are not selective. The most well-known phytocannabinoids are THC (Δ^8^-tetrahydrocannabinol) and CBD (cannabidiol). Research to date reports that both THC and CBD have an affinity for various types of endocannabinoid system receptors [5,6,7]. For example, cannabidiol is a non-competitive CB1 antagonist, CB2 inverse agonist, GPR55 and GPR18 antagonist, Peroxisome proliferator-activated receptor (PPAR-γ) agonist, α1, α3 glycine agonist, TRPM8 antagonist, and an agonist and antagonist of receptors of various types of serotonin 1A receptors (5-HT1A) [8,9,10,11]. The pharmacological effects of the interaction of Active pharmaceutical ingredients (APIs) with CB1 and CB2 receptors are the most investigated. The differences in the density of CB1 and CB2 receptor distribution also determine the activities within the central nervous system [12]. The pharmacological effect of cannabinoids results from modification of the signaling pathway of two G protein-coupled receptors (GPCRs): cannabinoid receptors 1 and 2 (CB1 and CB2). Activation of the CB1 receptor, which is expressed mainly in the central nervous system, is responsible for their psychotropic action, while CB2 receptors are found mainly in the immune system [13]. This distinct distribution of CB2 receptors in human tissues suggests that selective cannabinoids are promising APIs with anti-inflammatory, analgesic, and anti-neuroinflammatory actions [14]. The discovery of new selective cannabinoids can be streamlined with the application of in silico methods such as structure-based virtual screening (VS) [15,16,17] or ligand-based (quantitative structure-activity relationship (QSAR)) VS [18].

Machine learning-based QSAR models can be successfully applied in pharmaceutical research related to drug discovery [19], drug formulation [20], and pharmaceutical analysis [21,22]. The applicability of QSAR modeling to predict the selectivity of CB receptors has also been reported in the literature. The CB2-selective activity of 29 benzimidazole and benzothiophene derivatives was investigated with comparative molecular field analysis (CoMFA) and comparative molecular similarity indices analysis (CoMSIA) 3D-QSAR models [23] and showed an external predictive performance of R^2^ > 0.9. In the recent 4D-QSAR study involving molecular dynamics (MD), the modeling was performed on 29 structurally similar CB2 receptor inverse agonists [24]. The 4D-QSAR approach based on partial least squares (PLS) and multiple linear regression (MLR) resulted in Q^2^ = 0.719 and Q^2^ = 0.761 for the PLS and MLR models, respectively. The selectivity of 29 arylpyrazole derivatives was investigated with the application of 3D-QSAR/CoMFA analyses [25]. The QSAR model helped to identify the causes of CB1 selectivity by producing counter maps of affinity for both CB receptor subtypes. Nevertheless, the narrow applicability of these models due to similar scaffolds used as training examples may be a significant limitation when they are used in the prediction of novel chemotypes. In contrast, Floresta et al. conducted a 3D-QSAR study on a diverse dataset containing 312 molecules with reported experimental CB1 affinities and 187 molecules with reported CB2 affinities [26]. These models showed a predictive performance of Q^2^ = 0.62 and Q^2^ = 0.72 for the CB1 and CB2 QSAR models, respectively. The diversity of molecular structures in the training set also allowed Floresta et al. to perform VS for novel chemical scaffolds. The idea of the QSAR application for the identification of bioactive compounds in plant extract was also used by Labib et al. [27]. This VS study aimed to predict the activities of CB1 and opioid receptors among compounds isolated from *Pinus roxburghii* bark extract. The model of Labib et al. explained the synergistic anti-inflammatory action of *Pinus roxburghii* and provided information on the activity of several bioactive molecules identified in the extract.

Our study aimed to predict CB2-selectivity for molecules identified in *Cannabis Sativa* by using a validated QSAR model. This goal was achieved by the execution of several steps: the collection of diverse ligands’ structures with experimental data describing CB1 and CB2 affinity; data curation; conducting QSAR study for CB1 and CB2; and the prediction of the CB1 and CB2 affinity of phytochemicals in the *Cannabis Sativa*. So far, the lack of crystal structures in cannabinoid receptors has been a major obstacle in searching for new, selective cannabinoids. However, there have been attempts to predict binding modes of well-known actives of CB1 and CB2 using homology models of these receptors and molecular dynamics [17,28,29]. Recent advances in structural studies of cannabinoid receptors 6KPC [30], 6KPF [30], 6PT0 [31], and 5ZTY [32] provided new data that supplemented drug discovery studies [30]. In this study, we tested the applicability of available experimental structures of CB2, solved in both active and inactive conformations, in structure-based VS to search for novel or more CB2-selective ligands.

## 2. Results

### 2.1. Study Design

Data on the experimentally evaluated affinity of diverse compounds to CB1 and CB2 receptors and data on structures identified in *Cannabis Sativa* with unknown CB1/CB2 affinity were collected from publicly accessible online resources. Next, the data with known affinity values were curated and subsequently used for the development and validation of CB1 and CB2 QSAR models. The validated models were used for the prediction of the CB1 and CB2 affinity of *Cannabis Sativa* ingredients and the prediction of prospective CB2-selective Cannabis Sativa ingredients. The dataset collected for the QSAR study was used to evaluate the statistical characteristics of the discriminatory ability of CB1 and CB2 crystal structures in the docking study.

### 2.2. Data Curation

**Figure 1:**
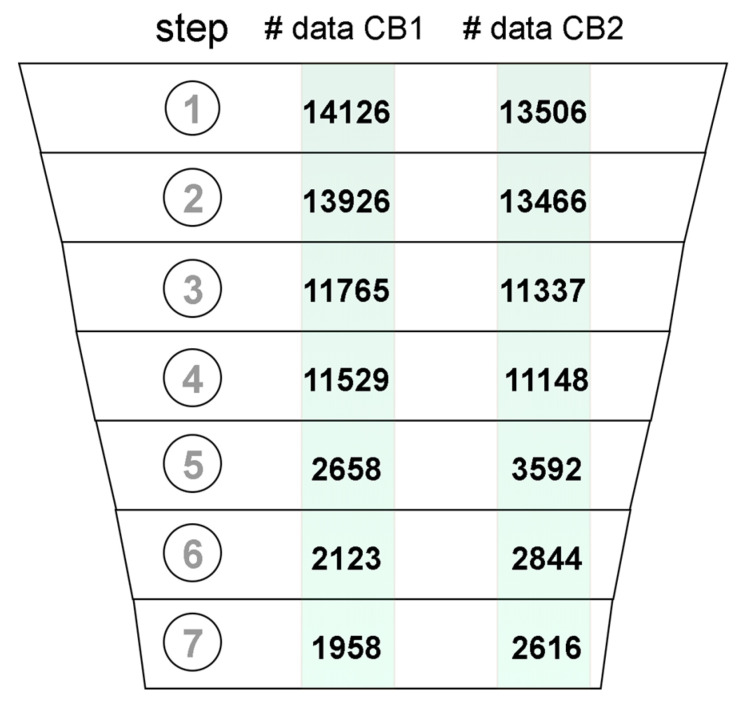
Data records that passed through the subsequent data curation steps: (**1**) data acquisition, (**2**) removing records with no SMILES included, (**3**) removing records with Confidence Score < 8, (**4**) keeping only records from large assays, (**5**) keeping only records with included Ki values, (**6**) duplicate merging, (**7**) removing molecules with the same feature vector.

Datasets of ligands with experimental affinities for CB1 and CB2 were joined with corresponding records of assay metadata from ChEMBL. The resulting dataset was composed of six columns containing: the structure of the molecule in simplified molecular-input line-entry system (SMILES) format, a standard type of reported value (i.e., Ki, IC50, etc.), reported value, the mathematical relation of the reported value to the experimentally measured value, the ID number of the source document with reported study, and the confidence score. The initial dataset described above was subjected to the removal of potentially unreliable data (Figure 1). The initial dataset included 14,126 records for CB1 and 13,506 records for CB2 (Figure 1.1). Records without reported SMILEs, which included metal complexes and polymers were excluded from the dataset (Figure 1.2). For the remaining records, the respective molecular structures were standardized and 2D coordinates were generated. We assumed that both the assays, which were carried out on a target protein or a homologous protein were reliable. Records meeting these criteria were annotated in ChEMBL with a confidence score of 9 and 8, respectively (Figure 1.3). Only activities measured in large and consistent assays were picked from the dataset (Figure 1.4).That is, an assay was considered as suitable for selection if it contained a consistent group of 10 or more molecules, all with the affinity measured. These large assays were identified based on the document ID reported in ChEMBL. In the next step, records with a reported affinity in any standard value type related to Ki were kept (Figure 1.5) and subsequently converted to pKi. Duplicates analysis (Figure 1.6) was conducted by comparing records InChIKeys. Duplicates were merged if the standard deviation of duplicate measurements was lower than 10% of the entire range of measurements. As a result, only one record was kept, with the pKi value averaged. Mordred descriptors and Morgan fingerprints were computed for each compound in the dataset and then concatenated to create a feature vector. Records with duplicate feature vectors were removed (Figure 1.7). These included, e.g., compounds differing only by chiral hydrogen atoms and those that could not be differentiated by the used descriptors and fingerprints.

### 2.3. Model Description

**Figure 2:**
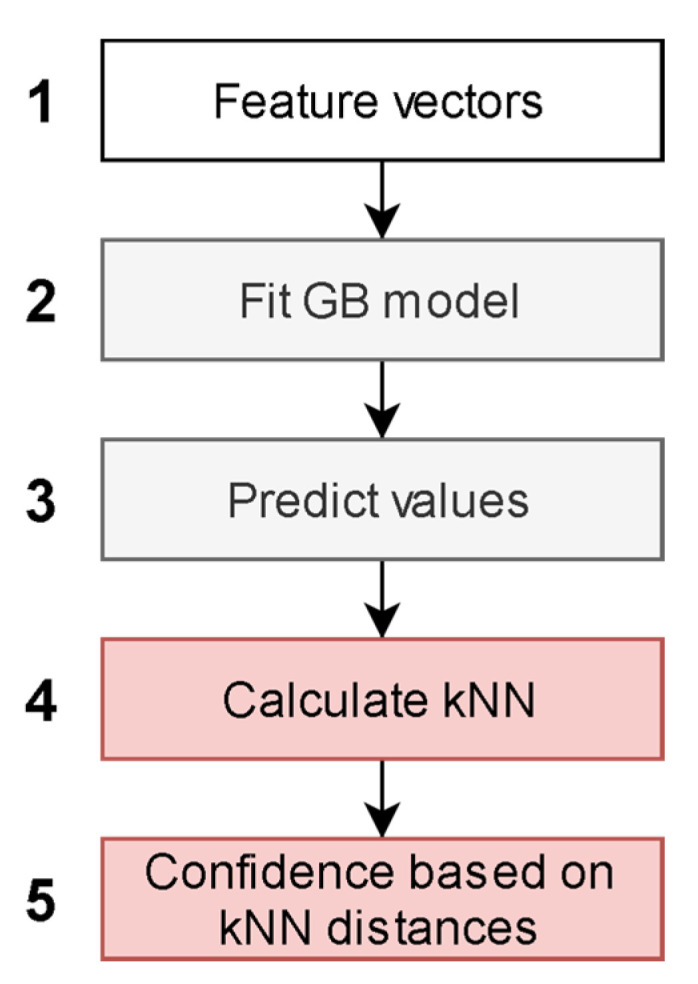
Machine learning-based quantitative structure-activity relationship (QSAR) model.

The independent CB1 and CB2 QSAR models were created according to the architecture presented in Figure 2. The input feature vectors for the machine learning algorithm were molecular descriptors and fingerprints of compounds from the training set (Figure 2.1). The feature vectors along with experimental CB1 and CB2 pKi values were used as a training set for the CB1 and CB2 QSAR models (Figure 2.2).

The value predicted in each leaf of decision trees in a gradient boosting (GB) ensemble was used for the embedding of a descriptor space (Figure 2.3). The embedded representation of the descriptor space was used to create a separate k-nearest neighbor (kNN) model for each receptor (Figure 2.4). The kNN algorithm was modified to predict an average pKi value of a compound only if all nearest neighbors of the query molecular structure were within a given distance. This maximal distance was used as an applicability domain threshold related to the confidence of prediction (Figure 2.5). Different thresholds were tested to assess their influence on the statistical characteristics of kNN models. We selected 20 thresholds as percentiles 5 to 100 with a step equal to 5% of the distribution of maximal Euclidean distances within kNN clusters obtained for the training set.

### 2.4. Model Validation

**Figure 3:**
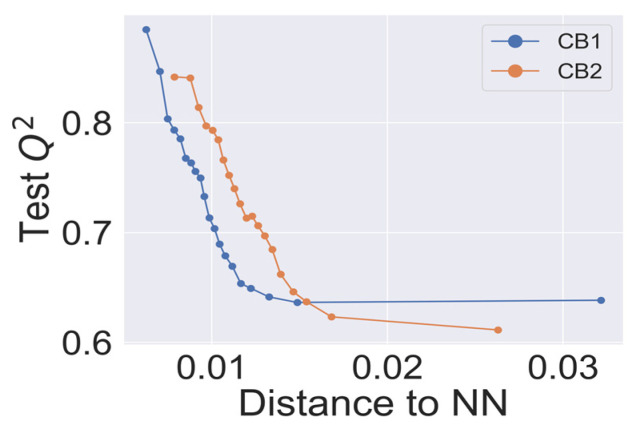
Q^2^—applicability domain threshold dependence for CB1 and CB2 k-nearest neighbor (kNN) models. Each point on the curves represents a Q^2^ calculated for external predictions for a cumulative fraction of molecular structures in the training dataset within a step of 5%.

In Figure 3, we present the dependence of cross-validated Q^2^ on the applicability domain threshold. The predictive performance showed consistent behavior, declining with an increased threshold. The best statistical characteristics Q^2^ > 0.8 for the CB1 and CB2 models was observed for the lowest thresholds (<0.01). Interestingly, kNN models with the highest threshold, which corresponds to the most inclusive applicability domain, still achieved the statistical characteristics Q^2^ > 0.6, as suggested in studies of QSAR best practices [33].

**Figure 4:**
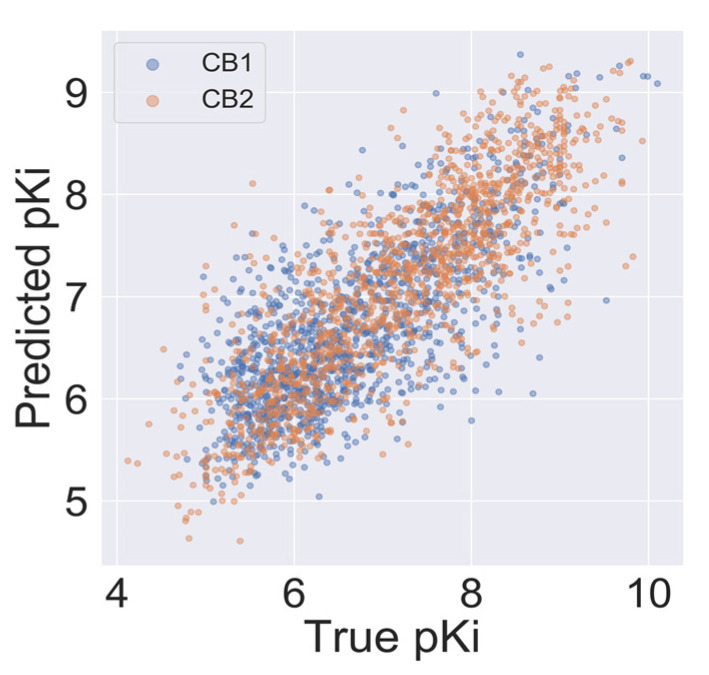
Autocorrelation of experimental and predicted pKi values for the CB1 model (blue) and the CB2 model (orange).

An autocorrelation plot (Figure 4) shows good agreement between out-of-sample predictions and experimental values for the majority of compounds, and importantly, for the CB2 selectivity ranges of pKi (pKi < 6 for CB1 and pKi > 6.5 for CB2). The majority of data points presented in Figure 4 are distributed between 6 and 8. Because machine learning techniques used in this study are interpolating techniques, we expected our model to overestimate pKi predictions at lower values and underestimate predictions at large ones.

### 2.5. Virtual Screening of Cannabis Sativa Phytochemicals

**Figure 5:**
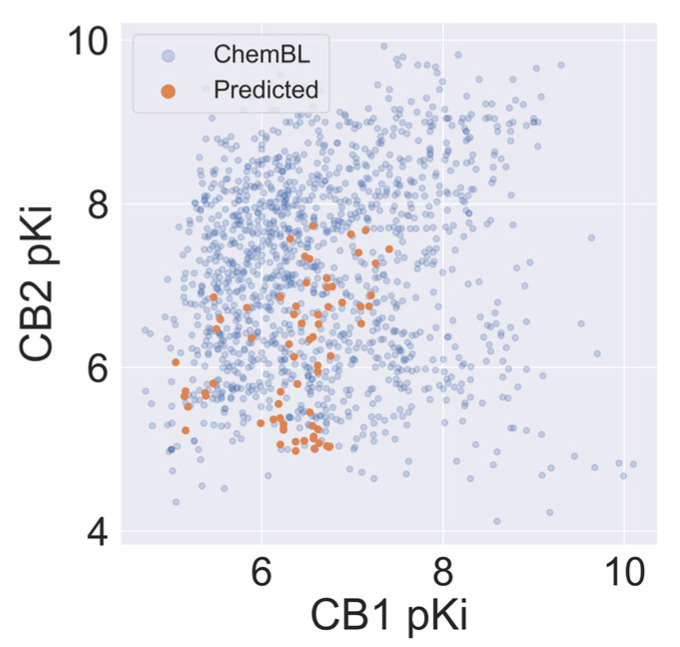
The correlation between affinity against CB1 and CB2 for compounds in *Cannabis Sativa* and compounds reported in ChEMBL.

Sixty-eight *Cannabis Sativa* phytochemicals with a molecular weight between 250D and 500D were subjected to VS using our validated CB1 and CB2 QSAR models. On average, the selectivity of compounds from *Cannabis Sativa* was less than for the ChEMBL-derived training set. Still, we observed a relatively high autocorrelation of the predicted CB1 and CB2 activity of *Cannabis Sativa* phytochemicals (see Figure 5) in comparison to the ChEMBL training set. The average absolute *d*pKi between CB2 and CB1 was 0.69 and 1.26 for molecules in *Cannabis Sativa* and molecules in the training set, respectively.

Despite relatively low average predicted selectivity of compounds in Cannabis Sativa, structures C1–C3 showing dpKi > 1 and a Tanimoto coefficient (TC) < 0.5 were identified (Table 1). Low TC values indicated a significant structural difference in the identified molecular structures in *Cannabis Sativa* compared to the respective most similar molecular structures in the training set.

### 2.6. CB2 Structure-Based Virtual Screening Results

**Figure 6:**
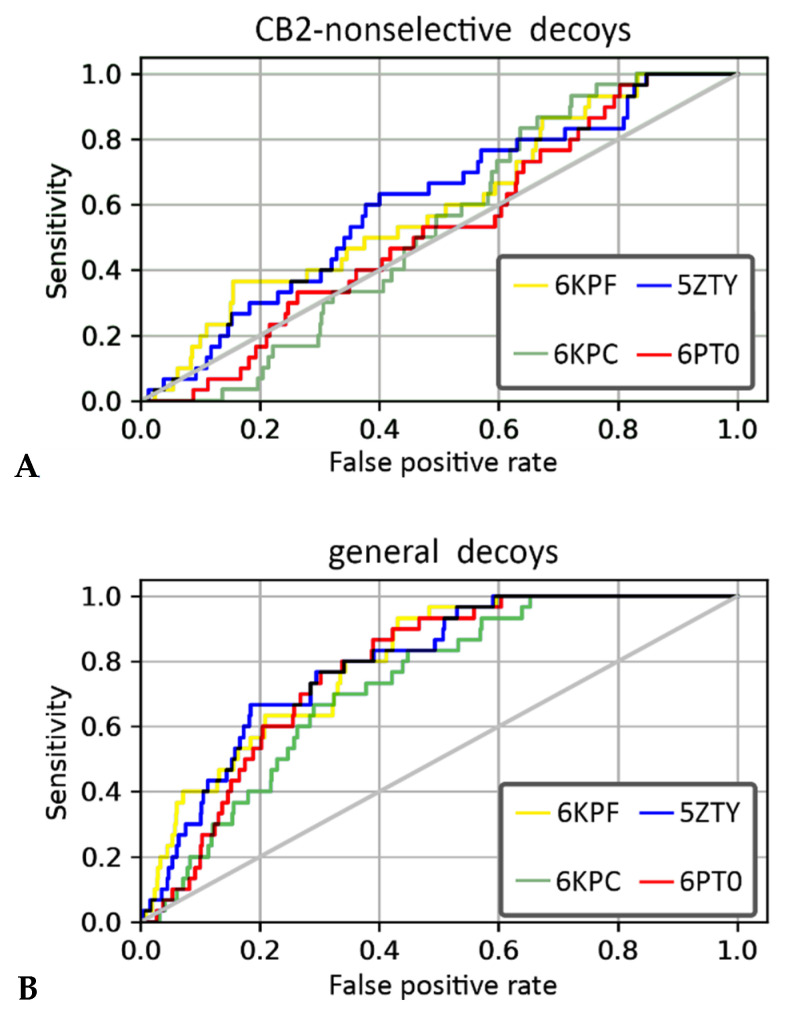
The receiver operating characteristic (ROC) curves obtained in the enrichment study using libraries including (**A**) CB2-selective and CB2-non-selective ligands and (**B**) CB2-actives and CB2-non-active ligands.

We performed VS using four PDB structures of the CB2 receptor (PDB id: 6KPF, 6KPC, 6PT0, and 5ZTY) and two compounds’ libraries. The library for the first compound was derived from the curated, ChEMBL training dataset prepared for our QSAR models and it included CB2-selective ligands expanded with CB2-non-selective ligands. For this compound library, 6KPF and 5ZTY structures achieved the highest area under the receiver operating characteristic curve (ROC AUC) value in the discrimination between CB2-selective ligands versus CB2-non-selective ones (see Table 2). The antagonist-bound, 5ZTY structure slightly outperformed 6KPF (agonist-bound), as observed in a detailed analysis of ROC curves obtained in our enrichment study (Figure 6). The second compound’s library used in our structure-based VS included the same CB2-selective ligands derived from the ChEMBL training dataset, named here with the term “actives”, and general diverse decoys that were generated with DUD-E [34], named here as “non-actives”. In VS against this second library, 6KPF and 5ZTY structures performed similarly, discriminating CB2-actives from non-actives at the level of 0.8 and 0.79, respectively (see Table 2, ROC AUC values). 6KPF slightly outperformed 5ZTY according to the ROC AUC characteristics and 6KPC performed the worst, however, the values were very close with no explicit difference between active and inactive CB2 structures. Our results showed that even slight differences between the crystal structures of the same receptor have an impact on the VS results. A similar conclusion was derived from a recent study on glucagon receptors, where an ensemble of receptor conformations generated in short MD simulations outperformed crystal structures in VS [35,36].

## 3. Discussion

The main goal of this study was to predict CB2-selective compounds among the phytochemicals that constitute *Cannabis Sativa*. We achieved this goal through the following steps: collecting a large set of experimental data from ChEMBL for the training set, data curation, model development and validation, and predicting the selectivity of phytochemicals from *Cannabis Sativa*. In parallel, we tested the applicability of recently released PDB structures of CB2 in structure-based VS by conducting an enrichment study. The dataset collected by our group was curated, which resulted in the removal of more than 90% of unreliable and inconsistent data. Despite the removal of this large fraction of data, the final dataset included 1958 records for CB1 and 2616 records for CB2. Notably, such a large dataset is sufficient for employing the machine learning approach. Much smaller datasets have been successfully used to develop QSAR models for CB1/CB2 studies [23,24]. Nevertheless, focusing on the dataset diversity in developing our QSAR model also created a challenge for predicting the activity of outlier compounds. We solved this problem by determining an applicability domain for each compound individually. Embedding the descriptor space of base GB models made it possible to assess the prediction confidence based on Euclidean distances of k-nearest neighbors. The GB algorithm was also successfully applied by *Ancuceanu* et al. [37] for cytotoxicity prediction. The performance of GB in the modeling of various QSARs was compared to other ensemble methods in a study by Kwon et al. [38].

Our approach was validated with the five-fold cross-validation protocol with resulting statistical characteristics of Q^2^ > 0.6 for the entire dataset and Q^2^ > 0.8 for the most confident predictions. Regarding small subsets with the highest confidence predictions, our model outperformed much more complex 3D and 4D QSAR models created for equally small sets of similar compounds [23,24]. We used our validated QSAR models for the prediction of the CB1 and CB2 affinities of phytochemicals from *Cannabis Sativa*. As a result, we identified three compounds C1–C3 (see Table 1) dissimilar to the training dataset with a TC < 0.3 compared to the respective most similar structure, and predicted pKi difference CB2 vs. CB1 (*d*pKi) ≥ 1. The common name of C1 is cannabispirol, and it is a compound isolated from Japanese domestic *Cannabis Sativa* [39]. C1 has been experimentally evaluated for antimicrobial activity [40] and was also tested in a study on targeting multidrug resistant mouse lymphoma cells [41]. Ilicic acid (C2) showed activity in G2/M cell cycle arrest of tumor cells [42]. Cannabichromente (C3) is a precursor for biosynthesis of various cannabinoids [43].

As for structure-based VS using PDB structures of CB2, we observed that these structures were able to discriminate not only CB2-actives from non-active ligands (DUD-E decoys) but also CB2-selective from non-selective ligands (the ChEMBL-derived dataset). The best results were obtained for 5ZTY and 6KPF structures, regardless of the activation state.

In this study, we identified potential selective cannabinoids among the constituents of *Cannabis sativa*. Our QSAR model identified three compounds of potential interest with significant dissimilarity to compounds already evaluated experimentally and reported in ChEMBL. The statistical characteristics of the developed QSAR models suggest a high probability of successful experimental validation, which we hope will attract attention from experimental groups interested in searching for CB2-selective ligands of natural origin.

## 4. Materials and Methods

### 4.1. Data Collection

Chemical structures from the ChEMBL database [44] with experimentally measured affinities for the CB1 and CB2 receptors were used as training data. The datasets were composed of 14,126 records for CB1 and 13,506 records for CB2. Compounds constituting the phytochemical profile of *Cannabis Sativa* were acquired from the Collective Molecular Activities of Useful Plants Database (CMAUP) [45]. The ChEMBL data was exported and downloaded in comma-separated values (CSV) format, while the CMAUP data was downloaded as an Structure Data Format (SDF) file.

### 4.2. Data Curation

The method of data curation followed a modified approach of Fourches et al. [46,47]. We modified the curation method by the addition of extra steps that were relevant for curating data acquired from ChEMBL accompanied by additional metadata specific to this database. Namely, we grouped experimental values provided their source studies were the same, and then removed groups with less than 10 records. We also assessed data reliability according to the source study confidence score as provided by ChEMBL. We then removed records with a confidence score lower than 8. A final dataset consisted of records with InChIKeys as molecular structure identifiers with associated standardized 2D structures of compounds and pKi values. RDKit was used for standardization and InChIKeys calculation [48]. The curated CB2 and CB1 datasets are available on the website [49].

### 4.3. Descriptor Calculation

The Mordred python library [50] was used to calculate 1613 2D standard descriptors and 213 3D descriptors. 3D descriptors were used to include information about chirality in ligand structures. Descriptors with variance less than 0.05 or containing invalid values were removed. The descriptors were concatenated with 1024-bit Morgan fingerprints with a radius equal to 3.

### 4.4. Machine Learning

A gradient boosting (GB) algorithm implemented in the Light Gradient Boosting Machines library [51] was used to create base models. The GB algorithm and its parameters were selected in an inner 5-fold cross-validation protocol based on grid search. Other algorithms including linear regression, partial least squares, and support vector machines from the scikit-learn library [52] were also tested. GB models for CB1 and CB2 were decision tree ensembles. The output of each tree in a boosted ensemble was used to create embedding of a descriptor space. A kNN algorithm with *k* = 3 was used to determine the applicability domain of the model based on the embedding. The applicability domain was defined by a threshold, which is a maximum allowed distance between a query molecular structure and nearest neighbors. Molecular structures with the Euclidean distance to nearest neighbors greater than a given threshold were considered as out of the applicability domain. Python code for the execution of trained models is provided in the Supplementary Materials.

### 4.5. Model Validation

Models were validated using a 5-fold cross-validation protocol. Out-of-sample predictions were used to calculate *Q*^2^ according to the following equation:

(1) $$Q^{2}=1-\frac{\sum_{n}{\left(Y_{i}-{\hat{Y}}_{i}\right)}^{2}}{\sum_{n}{\left(Y_{i}-{\bar{Y}}_{i}\right)}^{2}}$$

Here, *n* is the number of samples in the dataset, *Y_i_* is an experimental pKi of the ith sample, ${\hat{Y}}_{i}$ is a predicted value of pKi of the ith sample, and ${\bar{Y}}_{i}$ is a mean pKi value. We evaluated *Q*^2^ for 20 different applicability domain thresholds and considered *Q^2^* as a confidence measure for a model using a given threshold.

### 4.6. Prediction of CB2-Selectivity of Cannabis Sativa Ingredients

A curated dataset of *Cannabis Sativa* constituents reported in the CMAUP database [45] was tested against our QSAR CB1 and CB2 models. In such a way, we searched for selective molecules with *d*pKi ≥ 1, where *d*pKi was defined as *d*pKi = pKi_CB2_ − pKi_CB1_. For each predicted selective compound, a Tanimoto coefficient (TC) was calculated to assess the similarity of predicted compounds to ones already tested and used to exclude hits that were not novel with regard to training data. TC was computed using 1024-bit Morgan fingerprints with a radius equal to 3.

### 4.7. Structure-Based Virtual Screening

**Figure 7:**
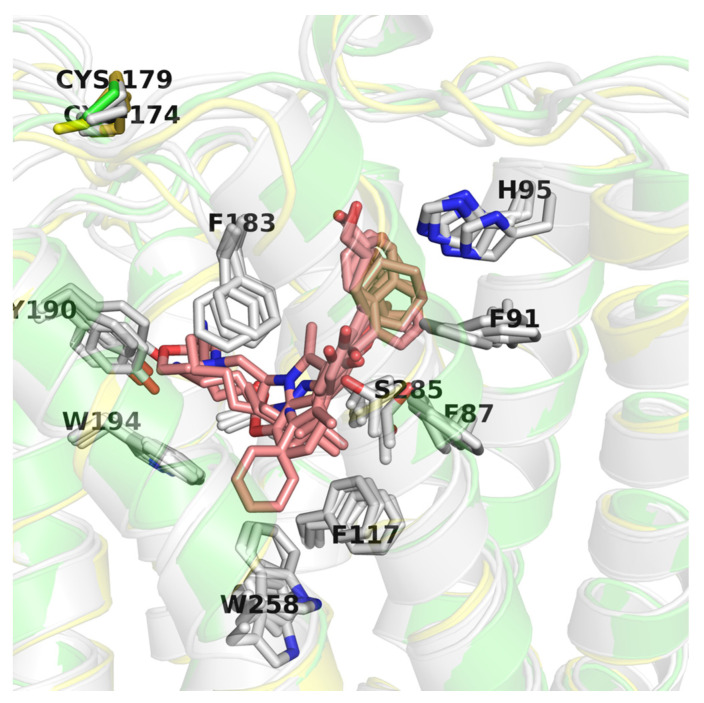
Comparison of CB2 structures available in the Protein Data Bank (PDB). Here, an inactive CB2 conformation (5ZTY) is shown in green and is the best performing in virtual screening (VS), active conformation (6KPF) is shown in yellow, and other CB2 active structures are shown in grey. Residues are labeled according to the 5ZTY numbering.

CB2 receptor structures (PDB id: 6KPC [30], 6KPF [30], 6PT0 [31], and 5ZTY [32]) were derived from the Protein Data Bank (PDB) [53]. This set included agonist-bound (6KPC, 6KPF, 6PT0) and antagonist-bound (5ZTY) structures corresponding to different activation states of the CB2 receptor (active and inactive, respectively). The CB2 structures used in this study were very similar to each other (average pairwise RMSD = 6.41, with standard deviation = 3.40), yet differing in TMH6 conformation (5ZTY and 6KPC vs. others) bending while interacting with a G protein complex. In all these structures ligands were located in the orthosteric binding site of CB2, although allosteric modulation has also been observed for these receptors [54]. The four PDB structures have nearly identical binding sites except for a few residues: W258 (W6.48), S285, and to a lesser extent: F87 (F2.57), F91 (F2.61), H95 (H2.65), F183 (EC2) (see Figure 7). W258 and F117 (F3.36) residues form a well-known Trp-Phe toggle switch changing its position on the receptor activation [17,30]. Interestingly, the position of W258 is slightly different not only in comparing the active vs. inactive conformations but also in three active conformations, e.g., moved away from the ligand (6PT0 vs. two others).

Receptor structures were processed with the Protein Preparation tool 2017-4, Schrodinger LLC, New York, NY, USA [55]. Ligands in PDB structures were used to determine the position of docking grids spanning over the whole CB2 active site. The prepared CB2 structures were used for screening against a CB2 actives library that included CB2-selective and CB2-non-selective compounds to assess the ability of these CB2 structures to discriminate between selective and CB2-non-selective ligands. This compound library was extracted from our ChEMBL training set (see Materials and Methods) and included compounds with pKi_CB1_ <= 5.5 and pKi_CB2_ > 7 for CB2-selective, while others were considered CB2-non-selective. The second round of VS was performed to test to what extent experimental CB2 structures were able to discriminate between CB2-actives and non-actives. Here, we used the compounds library that was generated using DUD-E [34] with ChEMBL-derived CB2-selective ligands (see above) used here as CB2 actives. Here, we discard CB2-non-selective ligands and we did not include them in the compound library. VS was performed with Glide 2017-4, Schrodinger LLC, New York, NY, USA [56] that followed the ligand preparation with Ligprep [57]. We computed enrichment factors (EF) and areas under the curve (AUC) for receiver-operator curves (ROC) using Maestro 2017-4, Schrodinger LLC, New York, NY, USA.

## 5. Conclusions

We would like to emphasize that our study aimed to perform an in silico study to identify prospective CB2-selective compounds from *C. Sativa* that could be confirmed by experimental studies in the future. This study shows the first robust CB1/CB2 QSAR models that are reproducible and applicable to a large variety of chemical scaffolds. Our developed model was trained on a large and diverse set of compounds that surpassed recent studies [26]. We based our method on machine learning, which utilized data from thousands of experimental studies on CB1/CB2 activities. Our study was been conducted without making common mistakes such as, e.g., not conducting data curation or its improper execution, lack of a validation protocol, applying inappropriate statistical characteristics for estimation of predictive performance, and finally, lack of applicability domain determination [58,59,60]. We also showed that the applicability domain estimation based on a gradient boosted model latent space allows the accurate prediction of the model confidence. What is more, we validated the estimation of the model confidence interval, which allows users to make conclusions based on the general statistical characteristics of a model and also on the validated confidence of a single prediction. No prior QSAR study on cannabinoids have showed these results.

The phytochemistry of *C. Sativa* has been described in many experimental and theoretical studies (see ElSohly et al. [2]). The analgesic effect of *C. Sativa* extracts has been known for many years [61]. Among the many experimental studies regarding cannabinoids, a few studies describing the three compounds selected by us deserve attention [39,40,41,42,43]. What is more, in the last year, many valuable and novel studies on *C. Sativa* applications in medicine have been published [62,63,64,65,66], thus, we hope that the exploration of the potential use of *C. Sativa* in pharmacotherapy will continue both experimentally and by using theoretical models.

## Figures and Tables

**Table 1:** Compounds from *Cannabis Sativa* that were predicted as CB2-selective and are also dissimilar to the ChEMBL training set.

Structure	Predicted Value		Similar Molecule for ChEMBL			Tanimoto Coefficient
	CB1 pKi	CB2 pKi	Structure	CB1 pKi	CB2 pKi	
C1 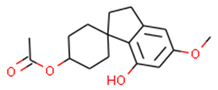 (4-hydroxy-6-methoxyspiro[1,2-dihydroindene-3,4’-cyclohexane]-1’-yl) acetate	5.46	6.87	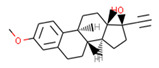 CHEMBL1201151	4.64	N/A	0.28
C2 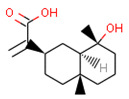 2-[(2R,4aR,8R,8aR)-8-hydroxy-4a,8-dimethyl-1,2,3,4,5,6,7,8a-octahydronaphthalen-2-yl]prop-2-enoic acid	5.57	6.59	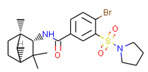 CHEMBL256753	6.95	8.03	0.18
C3 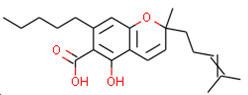 6-carboxy-2-methyl-2-(4-methylpent-3-enyl)-7-pentylchromen-5-olate	6.41	7.29	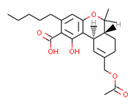	7.33	6.04	0.41

**Table 2:** Results of the enrichment study for CB2-receptor structures.

Metric	CB2-Non-Selective Decoys				General Decoys			
	6KPF	6KPC	6PT0	5ZTY	6KPF	6KPC	6PT0	5ZTY
EF 2%	0	0	0	1.7	3.3	0	0	3.3
EF 5%	0.67	0	0	1.3	4.7	1.3	1.3	3.3
EF 10%	1.7	0	0.33	1	4.0	2	1.7	3
ROC AUC	0.6	0.53	0.53	0.6	0.8	0.73	0.77	0.79

## Supplementary Materials

Supplementary materials can be found at https://www.mdpi.com/1422-0067/21/15/5308/s1.
